# Factors influencing self-efficacy for self-management among adult people with human immune deficiency virus on antiretroviral therapy in public hospitals of south-west Ethiopia

**DOI:** 10.3389/fpsyg.2024.1329238

**Published:** 2024-02-06

**Authors:** MisganaTesgera Abdisa, Bekan Gudeta Gindaba, Ebisa Zerihun

**Affiliations:** ^1^Department of Nursing, College of Health Science, Mattu University, Mattu, Ethiopia; ^2^School of Nursing and Midwifery, Institute of Health Science, Wallaga University, Nekemte, Ethiopia; ^3^Department of Nursing, College of Health Science, Oda Bultum University, Chiro, Ethiopia

**Keywords:** antiretroviral therapy, HIV, influencing, self-efficacy, self-management, Ethiopia

## Abstract

**Introduction:**

Self-management is crucial for effective HIV management, and self-efficacy is a mechanism for achieving it, but there is limited evidence on variables that affect self-efficacy. This study aimed to identify factors influencing self-efficacy for self-management among adults on antiretroviral therapy in resource constraint settings.

**Methods:**

A cross-sectional study was conducted among 422 adult people on antiretroviral therapy in southwest Ethiopia from March to April 2022. Face-to-face interviews were used to gather data using a structured questionnaire on the self-efficacy measure. The data were then imported into Epi Data version 4.2 and exported to SPSS version 26. Descriptive statistics, independent tests, one-way analysis of variance, Pearson correlation, and multivariate linear regression were used to analyze the data. The predictors with *p*-value of less than 0.05 were declared statistically significant.

**Results:**

A total of 413 adults on antiretroviral therapy were interviewed with response rate of 97.9%. The total mean score of self-efficacy for self-management was 15.12 (±2.22) out of 24. Higher age, gender of the female, divorced, duration of diagnosis, and drug side effects were negatively predictors of low self-efficacy. Higher schooling, urban residence, better income, and the use of reminders positively influenced self-efficacy for self-management.

**Conclusion:**

The study found low self-efficacy among adults on antiretroviral therapy and higher age, female gender, HIV duration, and presence of drug side effects were associated with lower self-efficacy, while higher schooling, better income, and use of reminder use were associated with higher self-efficacy for self-management. Further research is needed to determine the causal relationship between these variables and self-efficacy.

## 1 Introduction

According to the global HIV statistics report of 2020 worldwide, the number of persons living with Human Immunodeficiency Virus (HIV) was approximately 37.7 million; of these, 36 million were adults, which account for approximately two-thirds of the total living with HIV (FACT SHEET 2021, 2021). The HIV/AIDS pandemic has significantly impacted sub-Saharan Africa (FMOH, 2020), with Ethiopia alone housing approximately 620,000 people with HIV/AIDS (Girum et al., 2018). The advancements in HIV/AIDS treatment have transformed the experience of adult HIV patients from acute to chronic disease, encouraging self-management practices (Swendeman et al., 2009; Schulman-Green et al., 2012).

Enhancing self-efficacy is thought to be one way to improve patient self-management, which is crucial for individuals living with HIV who are receiving therapy in order to maintain their health and well-being (Areri, 2021). Self-efficacy for self-management is the capacity of an individual to plan and carry out a course of action to achieve the intended objectives (Bandura and Wessels, 1994). Increased self-efficacy improves well-being and self-management practice participation. For many chronic health disorders, including HIV, self-efficacy has a positive influence on self-management. Improving self-efficacy for self- management is crucial for enhancing quality of life, achieving optimal medication adherence, and participating in self-efficacy. By improving self-efficacy, people can improve their health behaviors and self-management. This can lead to better health outcomes and a higher quality of life (Shakya, 2018; Areri et al., 2020; Areri, 2021).

Improving individuals living with HIV on ART’s self-efficacy is essential to fostering their self-belief and confidence to carry out recommended self-management practices. Research indicates that the level of self-efficacy in individuals living with HIV has been found to positively influence their overall self-management (Adefolalu et al., 2014). Better experiences with self-management and better adherence to therapy are associated with higher levels of self-efficacy (Huang et al., 2013; Allegrante et al., 2019). Additionally, research has shown that raising the self-efficacy of adults living with HIV (ALWHIV) can facilitate resource mobilization, improve symptom management, and increase their ability to tolerate discomfort (Cha et al., 2008). Thus, higher levels of self-efficacy can both improve adherence to antiretroviral therapy and lower the burden of HIV infection. Therefore, it is critical to comprehend the variables that affect a person’s confidence in their ability to manage themselves in order to provide tailored help (Aregbesola and Adeoye, 2018; Adamu et al., 2020; Maay and Maryorita, 2020; Jadgal et al., 2022).

Studies have been conducted in several countries to determine the influencing factors of self-efficacy for chronic condition self-management with a similar instrument. However, the factors observed to influence self-efficacy for self-management varied across the studies. For instance, sociodemographic variables including monthly income, age, education level, job, gender, living area (Shakya, 2018; Areri, 2021), and treatment-related variables, like having adverse medication reactions (Wang et al., 2016; Areri et al., 2020) and social support (Criswell et al., 2010), were identified in previous research. In addition, other studies show that drug adherence is linked to self-efficacy, while drug-related side effects negatively impact self-management self-efficacy (Zhang et al., 2016; Okuboyejo et al., 2018). By targeting the factors that predict self-efficacy, interventions can help people improve their health behaviors and self-management.

However, there is limited evidence on factors affecting self-efficacy for adult self-management of antiretroviral therapy in developing countries, particularly in Ethiopia, whereas medical intervention and self-efficacy for self-management are scientifically recommended for controlling HIV-related complications, particularly in resource-constrained countries, such as Ethiopia. This study aimed to identify factors influencing self-efficacy for self-management among adults on antiretroviral therapy in public hospitals in south-west Ethiopia.

## 2 Materials and methods

### 2.1 Study design and area

An institutional-based cross-sectional study was conducted in three public hospitals in south-west Ethiopia, namely Mettu Karli Compressive Specialized Hospital, Jimma Medical Center, and Bedelle General Hospitals, from March to April 2022. Mettu Karli Compressive Specialized Hospital, Jimma Medical Center, and Bedelle General Hospitals provided ART services to 1,480, 2,400, and 987 adults with HIV, respectively.

### 2.2 Study population

All adults of ≥18 years living with HIV and enrolled in ART care in the three public hospitals were included in study population.

### 2.3 Inclusion and exclusion criteria

The study included adults (≥18 years) living with HIV on ART for at least 6 months. The study excluded those critically ill patients who were unable to respond and did not volunteer to sign a consent form.

### 2.4 Sample size determination and procedure

The sample size for this study was calculated using a single population proportion with a 95% confidence level, 5% margin of error, and 50% self-efficacy for self-management since there was no previously published research conducted in this study area, and the calculated sample size was 384. By considering the 105 of non-response rate, the final sample size was 422. This is a relatively large sample size, and it should be sufficient to detect a statistically significant difference. However, the effect size is small, and the data are relatively variable. This means that it may be difficult to detect a statistically significant difference.

The study involved a proportional allocation of a sample size to each public hospital, based on a list of adults in the ART registration book, and computer-generated simple random sampling was used to select the participants. Invitations to participate in the study were given to participants during standard clinic visits.

### 2.5 Measurement

To collect participant demographic data, such as age, educational attainment, place of residence, marital status, and monthly household income, the research team created a demographic survey. Data on HIV, such as the number of years from HIV diagnosis and treatment adverse effects, was also gathered.

The study assessed self-efficacy for self-management using an HIV self-management scale to self-efficacy for SM, with participants rating their self-efficacy on eight items; each scored using a three-point Likert scale. The total mean score was calculated by obtaining the sum of all items. For this, self-efficacy for self-management range 8 to 24, with higher scores indicating better self-efficacy (Wallston et al., 2011).

The study assessed HIV-positive status disclosure through self-reports of participants who disclosed their HIV status to others, including sexual partners, family, friends, or others (Melis Berhe et al., 2020).

### 2.6 Data collection tool and procedure

Data were collected using a structure questionnaire through a face to face interview in a quiet room. The questionnaires assess demographic and medical factors related to HIV, including treatment duration, drug use, and side effects, and promote social facilitation through HIV disclosure and reminders. A pretest was conducted on 5% of the total sample size outside the study area at Nekemte Comprehensive Specialized Hospital to ensure that questionnaires were appropriate and necessary modifications were made. The reliability of the tool was evaluated using Cronbach’s alpha, resulting in a score of 7.25.

Data were gathered by experienced nurses with orally administered survey tool from March to April 2022 at follow-up ART clinic. Data collectors underwent 1-day training on data collection procedures, and investigators regularly checked the completeness of the questionnaires.

### 2.7 Data processing and analysis

The collected data were checked for completeness, coded, and entered into Epi-data version 4.2 and then exported to statistical package for social science (SPSS) version 26 for statistical analysis. The study participants were characterized using descriptive analysis, which was then summarized using frequency and mean. The study utilized independent t-tests and one-way ANOVA to analyze the mean difference between self-efficacy for self-management and each independent variable. The relationship between self-efficacy for SM and each variable was identified using a Pearson correlation. Multivariate linear regression analysis was conducted to identify the factors that influence self-efficacy for self-management. Statistical significance was determined for independent variables with a *p*-value of less than 0.05.

### 2.8 Ethical approval and consent participate

The study received ethical approval from the research ethical review Committee College of Health Sciences at Mattu University and permission from Mettu Karli Compressive Specialized Hospital, Jimma Medical Center, and Bedelle General Hospitals. Participants provided written informed consent were informed about the study’s purpose, right to withdraw, and privacy. The procedures followed the Helsinki Declaration, ensuring confidentiality and privacy.

## 3 Results

### 3.1 Sociodemographic characteristics of the participants

A total of 413 adult individuals on antiretroviral therapy were interviewed, with a response rate of 97.9%. The mean age of participants was 40.09 (SD ± 1,234) years. Out of 413 respondents, 222 (53.8%) were women, while 182 (44.1%) were Orthodox religion followers. The study found that 207 (50.1%) of participants were single, 133 (32.2%) were educated in primary school, and 211 (51.1%) were unemployed. Among the majority of respondents, 280 (67.8%) were urban dwellers. The mean monthly income of participants was 1652.54 Ethiopian Birr (Table 1).

**Table 1:** Sociodemographic characteristics of adult on ART in Public Hospitals at south-west Ethiopia, 2022 (*n* = 413).

Variables		Self-efficacy for self-management			
	**Categories**	***N* (%)**	**M** ± *SD*	**Test statics**	***P*-value**
Mean age of participants (M ± SD)		40.09 ±12.34			
Gender	Male	191(46.2)	15.47 ±2.14	2.961	0.003*
	Female	222(53.8)	14.84 ±2.24		
Religion	Orthodox	182(44.1)	15.45 ± 2.15	9.168	<0.001*
	Muslim	150(36.3)	14.40 ± 2.42		
	Protestant	59(14.3)	15.68 ± 1.62		
	Other, specify	22(5.3)	15.86 ± 1.32		
Marital status	Single	207(50.1)	15.01 ± 2.54	3.841	0.010*
	Married	114(27.6)	15.02 ± 1.81		
	Divorced	58(13.6)	14.96 ± 1.86		
	Widowed	36(8.7)	16.31 ± 1.55		
Educational level	Illiterate	122(29.5)	14.86 ± 2.31	7.163	<0.001*
	Primary school	133(32.2)	14.73 ± 1.66		
	Secondary school	80(19.4)	15.24 ± 2.82		
	College and above	78(18.9)	16.08 ± 1.93		
Job status	Unemployed	211(51.1)	14.92 ± 2.15	1.919	0.148
	Private employed	145(35.1)	15.28 ± 2.49		
	Government employed	57(13.8)	15.46 ± 1.54		
Living area	Rural	133(32.2)	13.98 ± 1.85	−8.125	<0.001*
	Urban	280(67.8)	15.66 ± 2.18		
Monthly average income (Ethiopian birr)		M ± SD 1652.54 ±1228.32			

^*^Statistically significance mean difference at *p* < 0.05.

### 3.2 Medical and social facilitation characteristics of the participants

The mean durations since being diagnosed with HIV status-positive and taking HIV treatment were 3.04 $\left(\pm 1.43\right)\;\mathrm{years}$ and 26.95 $\left(\pm 9.37\right)\mathrm{months}$ , respectively. Over half of the participants, 274 (66.3%) used one type of HIV drug, while 315 (76.3%) did not complain about side effects. More than half of the participants, 223 (56.4%) and 253 (61.3%) did not use reminders for HIV treatments and disclosed their HIV status, respectively (Table 2).

**Table 2:** Medical and social facilitation characteristics of adults on ART in Public Hospitals at South West Ethiopia, 2022 (*n* = 413).

Variables		Self-efficacy for self-management			
	**Categories**	***N* (%)**	**M** ± *SD*	**Test statics**	***P*-value**
Duration of diagnosis (M ± SD)		3.04 ±1.43			
Duration of treatment (M ± SD)		26.95 ±9.37			
Number of HIV drugs used	One type	274(66.3)	15.43 ± 2.25	8.386	<0.001*
	Two types	55(13.3)	14.38 ± 1.48		
	More than two	84(20.3)	14.60 ± 2.31		
HIV drug side effects	Yes No	98(23.7) 315(76.3)	16.01 ± 1.81 14.84 ± 2.26	5.236	<0.001*
Use of reminders	Yes No	180(43.6) 223(56.4)	15.50 ± 2.67 14.83 ± 2.14	3.085	0.002*
HIV disclosure status	Yes No	160(38.7) 253(61.3)	14.90 ± 2.06 15.26 ± 2.30	−1.656	0.099

M, mean; SD, standard deviation. ^*^Statistically significance mean difference at *p* < 0.05.

### 3.3 Self-efficacy for HIV self-management of the participants and its correlate

The total score of self-efficacy for self-Management was 15.12 (SD = ±2.22) out of 24. More than half, 222 (53.8) of the respondents agreed with the statement says that “I find my efforts to change things I do not like about my HIV infection are ineffective.” However, approximately 207 (50.1%) and 184 (44.6) of the participants disagreed with the statements: I am able to manage things related to my HIV infection as well as most other people and I handle myself well with respect to my HIV infection, respectively, (Table 3).

**Table 3:** Respondents’ scores on self-efficacy for HIV self-management in Public Hospitals at South West Ethiopia, 2022 (*n* = 413).

**Questions**	**Response**		
	Disagree	Neutral	Agree
	*N* (%)	*N* (%)	*N* (%)
It is difficult for me to find effective solution for problems with managing my HIV infection	78(18.4)	179(43.3)	158(38.3)
I find my efforts to change things I do not like about my HIV infection are ineffective	83(20.1)	108(26.2)	222(53.8)
I handle myself well with respect to my HIV infection	184(44.6)	62(15)	167(40.4)
I succeed in the projects I under taken to manage my HIV infection	176(42.6)	106(25.7)	131(31.7)
I am able to manage things related to my HIV infection as well as most other people	207(50.1)	42(10.2)	164(39.7)
Typically my plan for managing my HIV infection do not work out well	130(31.5)	129(30.2)	154(37.3)
No Matter how hard I try, managing my HIV infection does not turn out the way I would like	113(27.4)	178(43.1)	122(29.5)
I am generally able to accomplish my goals with respect to my HIV infection	138(33.4)	141(34.1)	134(32.4)
**Mean total score of self-efficacy for self-management**		**15.12 ± 2.22**	

### 3.4 Correlation analysis of study participants

Higher age (*r* = 0.221, *p* = 0.001), being single marital status (*r* = 0.111, *p* = 0.024), educational level (*r* = 0.192, *p* = 0.001), areas of residence (*r* = 0.354, *p* = 0.001), income (*r* = 0.116, *p* = 0.018), duration of diagnosed HIV status as positive (*r* = 0.205, *p* = 0.001), and duration since starting of HIV treatment (*r* = 0.317, *p* = 0.001) were positively correlated with self-efficacy for self-management. However, gender (*r* = −0.145, *p* = 0.003), number of HIV drug used (*r* = −0.174, p = 0.001), drug side effects (*r* = −0.224, *p* = 0.001), and use of reminders (*r* = −0.15, *p* = 0.002) were negatively correlated with self-efficacy for self-management.

### 3.5 Factors influencing self-efficacy for self-management

To identify factors influencing self-efficacy for self-management among adults on ART, candidate predictor variables on bivariate analysis (correlation and simple linear regressions) with *p*-value of less than 0.05 entered into multivariate linear regression. The overall model was significant with *F*(11,401) = 21.566, *p* < 0.001, *R*^2^ = 0.372, Adjusted*R*^2^ = 0.354. The model explains 35.4% of the variance in self-efficacy for SM.

When the variance explained by all other variables in the model was controlled, age (*β* = −0.394, *p* < 0.001), gender of the female (*β* = −0.274, *p* < 0.001), being divorced (*β* = −0.499, *p* < 0.026), and the presence of drug side effects (*β* = −0.192, *p* < 0.001) showed significant and negatively influencing self-efficacy for self-management. However, higher educational level (*β* = 0.212, *p* < 0.001), being urban residence (*β* = 0.605, *p* < 0.001), duration since diagnosed HIV as positive (*β* = 0.499, *p* < 0.001), and use of reminder (*β* = 0.237, *p* < 0.001) showed significant and positively influencing self-efficacy for self-management (Table 4).

**Table 4:** Multivariate regression of factors influencing self-efficacy for self-management among adults on ART in Public Hospitals at south-west Ethiopia, 2022 (*n* = 413).

**Variables**	***B***	**SE**	***Β***	**95%CI for *B***		***P*-value**
				**LL**	**UL**	
Constant	17.124	0.949		15.258	18.989	0.001
Age(mean)	−0.071	0.011	−0.394	−0.093	−0.049	0.001*
Gender(ref. Male)	−1.218	0.271	−0.274	−1.752	−0.685	0.001*
Marital status(ref. single)	−0.266	0.119	−0.117	−0.499	−0.033	0.026
Educational level(ref. illiterate)	0.433	0.105	0.212	0.227	0.640	0.001*
Residence(ref. rural)	2.866	0.266	0.605	2.342	3.389	0.001*
Income(mean)	0.001	0.001	0.259	0.000	0.0001	0.001*
Duration of diagnosis(mean)	0.771	0.135	0.499	0.505	1.036	0.001*
Duration of treatment(mean)	−0.093	0.021	−0.393	−0.134	−0.052	0.001*
Number of drugs used	0.134	0.152	0.049	−0.164	0.433	0.378
Drug side effects (ref. Yes)	−1.000	0.246	−0.192	−1.483	−0.517	0.001*
Use of reminders (ref. yes)	1.059	0.198	0.237	−1.449	−0.670	0.001*
*R*^2^ (Adjusted *R*^2^) 0.372 (0.354) *F*(df) 21.566(11,401) *p* < 0.001 Durbin Watson 2.16						

^*^Statistically significance mean difference at *p* < 0.05.

## 4 Discussion

The study aimed to understand the factors influencing self-efficacy for SM among adult individuals in south-west Ethiopia, where HIV prevalence is high and healthcare access is limited. Self-efficacy is a key factor in health behavior change. People who believe that they are capable of changing their behavior are more likely to do so. The study found that adult people on antiretroviral therapy had a mean self-efficacy score of 15.12 (±2.22). The finding of this study was lower than the studies conducted in the north-west Ethiopia (Areri, 2021), Korea (Yoo et al., 2011), China (Huang et al., 2013), Indonesia (Maay and Maryorita, 2020), and United States of America (Lee, 2017). The findings of the study may differ due to variations in self-efficacy instruments, socioeconomic and demographic characteristics, health policy differences, study settings, and sample size. Moreover, health institutions should be focusing on psychosocial interventions that increase self-efficacy and can help people develop the skills and confidence they need to make healthy changes in their lives and cope with the challenges of living with HIV (Patrão et al., 2021).

The study found a positive correlation between age, education level, and self-efficacy consistent with previous research in Korea (Yoo et al., 2011). Adult HIV-positive individuals with higher education levels may possess information on living with the virus that can enhance their sense of self-efficacy for self-management by enabling them to stay informed about managing side effects and overcoming prejudice and stigma.

Our results showed that more income was linked to higher levels of self-efficacy for self-management. This outcome is in line with the findings of other previously published investigations in north-west Ethiopia (Areri et al., 2020). This is suggested that participants may have better incomes to meet their basic needs, potentially boosting their self-efficacy for SM. However, the study reveals that 51.1% of ALWHIV participants lack regular jobs, potentially contributing to low income. While short-term income modification is challenging, creating job opportunities could improve long-term income and self-efficacy for SM.

The study reveals that the use of reminders can enhance self-efficacy in self-management. These findings are consistent with those conducted in China (Wang et al., 2019). Moreover, to increase self-efficacy for self-management, a system for maximizing the use of reminders via phone calls, texts, online help, and other facilitation techniques must be developed.

In this study, duration of HIV diagnosed as positive and treatments were positively correlated with self-efficacy for self-management, which needs further studies to support the results of these findings. Our study revealed that drug side effects were negatively correlated with self-efficacy for self-management. The findings of this study were supported by a previous study in China (Huang et al., 2013). Drug side effects significantly impact HIV adults’ confidence in ART, requiring care providers to assess and support patients, teaching about side effect management and enhancing self-efficacy for self-management (Zhang et al., 2016). Implementing a structured self-management model at each point of care can help HIV care providers assess side effects, discuss causes and interventions, and enhance patients’ self-efficacy for health outcome management.

This study identified being rural residence positively influencing self-efficacy for self-management among adult people on antiretroviral therapy. This study’s results align with previous research conducted in north-west Ethiopia (Areri et al., 2020). The findings are not found in other studies (Zhang et al., 2016) and may be specific to the Ethiopian context. Ethiopia’s rural communities face low literacy, inadequate HIV treatment awareness, communication infrastructure issues, and low socioeconomic status. Limited information access and potential stigma can negatively impact self-management in rural communities, but continuous HIV education could help reduce these issues (Feyasa et al., 2022).

Overall, the results of this study point to the need for intervention to raise the self-efficacy of this population across all modifiable parameters that affect self-efficacy for self-management. Identifying and addressing issues connected to experiencing drug side effects may be made easier with the systematic and routine application of this strategy during follow-up visits. However, governments must act persistently to alter community attitudes around HIV and socioeconomic status and educational attainment.

This study has some limitations. First, due to this study was cross-sectional design, which makes it difficult to assess cause-and-effect relationships and capture longitudinal trend. In addition, we acknowledge that the power of our study is limited by the small effect size and the variable data. Second, patient self-reports and in-person interviews were used to gather the data, which could have been impacted by potential reporter bias because of social desirability. Third, although the translated instrument has been evaluated and found to be useful in other settings, its validity in the Ethiopian context has not been investigated.

## 5 Conclusion

This study showed that self-efficacy for self-management of adult on antiretroviral therapy was low. Ages, being female, divorced, duration of diagnosed, and experiencing drug side effects were negatively correlated with self-efficacy. Being higher educational level, urban residence, better income, duration of treatments, and the use of reminders were positively influencing self-efficacy for self-management. Moreover, health institution should focus on psychosocial interventions that increase the level of self-efficacy of people at risk of HIV because these interventions can help people to make healthy changes in their lives, cope with the challenges of living with HIV, and adhere to ART. Future longitudinal studies should investigate causal inferences and ensure the reliability of our findings.

### 5.1 Implications for practice

HIV care professionals should evaluate SM self-efficacy on a regular basis, offer constructive criticism, and encourage adults living with HIV to improve their SM self-efficacy. Patients should be informed about potential HIV treatment side effects and how to manage them. To improve adults living with HIV self-efficacy in SM, use intervention like self-management model during follow-up visits, increase patient belief in self-efficacy, and work on enhancing stigma and discrimination awareness particular in rural communities. Health institution should focus on psychosocial interventions that increase the level of self-efficacy of people at risk of HIV because these interventions can help people to make healthy changes in their lives, cope with the challenges of living with HIV, and adhere to ART. In order to alter community attitudes regarding HIV, poverty, and educational attainment, policymakers must take persistent action, focusing on creating awareness and enable them to earn an adequate income for adults living with HIV to improve self-efficacy.

## Data availability statement

The data that support the findings of this study are available from the corresponding author upon reasonable request.

## Ethics statement

The studies involving humans were approved by research ethical review committee of Mattu University, College of Health Sciences. The studies were conducted in accordance with the local legislation and institutional requirements. The participants provided their written informed consent to participate in this study.

## Author contributions

MA: Conceptualization, Funding acquisition, Investigation, Methodology, Project administration, Resources, Software, Supervision, Visualization, Writing – original draft, Writing – review & editing. BG: Conceptualization, Data curation, Formal analysis, Investigation, Resources, Supervision, Visualization, Writing – original draft, Writing – review & editing. EZ: Conceptualization, Formal analysis, Investigation, Methodology, Software, Validation, Visualization, Writing – original draft, Writing – review & editing.
